# Cerebral Mechanism of Tuina on the Descending Pain Inhibitory System in Knee Osteoarthritis: Protocol for a Randomized Controlled Trial

**DOI:** 10.2196/52820

**Published:** 2024-02-12

**Authors:** Hui Xu, Zheng Wang, Zhen Wang, Hang Zhou, Juan Guo, Wanyu Li, Yunfeng Zhou

**Affiliations:** 1 School of Acupuncture-moxibustion and Tuina Henan University of Chinese Medicine Zhengzhou China; 2 Tuina Department The Third Affiliated Hospital of Henan University of Chinese Medicine Zhengzhou China

**Keywords:** brain, knee osteoarthritis, magnetic resonance imaging, pain, Tuina

## Abstract

**Background:**

Knee osteoarthritis (KOA) is reputedly the most common musculoskeletal disease of the lower limbs and the main cause of pain and disability among older individuals. Pain is the most significant and widespread symptom of KOA. The descending pain inhibitory system has a cardinal role in normal pain consciousness, and its malfunction may be one of the pathophysiological mechanisms in KOA. Crucially, the rostral ventromedial medulla (RVM) and periaqueductal gray (PAG), as important components of the descending pain inhibitory system, directly modulate the activity of the spinal neurons involved in pain transmission. Tuina, a manual therapy, is effective and safe for reducing clinical symptoms of KOA; however, the mechanism that influences pain through the descending pain inhibitory system in KOA is unclear.

**Objective:**

This study aims to investigate the modulatory implications of Tuina on the RVM and PAG, which have critical roles in the descending pain inhibitory system in patients with KOA.

**Methods:**

This randomized controlled parallel trial will be conducted at the Tuina Clinic of the Third Affiliated Hospital of Henan University of Chinese Medicine (Zhengzhou, China). Patients with KOA will be randomly assigned (1:1) to 6 weeks of health education or Tuina. All patients in both groups will accept a resting-state functional magnetic resonance scan at the beginning and end of the experiment, and the resting-state functional connectivity and the voxel-based morphometry analysis will be performed to detect the RVM and PAG function and structure changes. The clinical outcome assessments will be (1) the pressure pain thresholds, (2) the Numerical Rating Scale, (3) the Hamilton Depression Scale (HAMD), and (4) the Western Ontario and McMaster Universities Osteoarthritis Index (WOMAC). Considering that this trial is a study of resting-state functional magnetic resonance imaging technology, resting-state functional connectivity and voxel-based morphometry are the primary outcomes, and clinical outcome assessments are secondary outcomes. Adverse events will be documented and assessed throughout. All main analyses will be carried out on the basis of the intention-to-treat principle. The outcome evaluators and data statisticians will be masked to the treatment group assignment to reduce the risk of bias.

**Results:**

This trial was approved by the ethics committee of the Third Affiliated Hospital of Henan University of Chinese Medicine. Enrollment began in December 2023, and the results of this trial are expected to be submitted for publication in May 2025.

**Conclusions:**

This trial will identify a possible relationship between function and structure changes of RVM and PAG and the improvement of clinical variables, elucidating the effect of Tuina on the descending pain inhibitory system of patients with KOA. This trial will provide much-needed knowledge for Tuina for patients with KOA.

**Trial Registration:**

Chinese Clinical Trial Registry ChiCTR2300070289; https://www.chictr.org.cn/showproj.html?proj=182570

**International Registered Report Identifier (IRRID):**

PRR1-10.2196/52820

## Introduction

Knee osteoarthritis (KOA) is reputedly the most common musculoskeletal disease of the lower limbs and the main cause of pain and disability among older individuals. Approximately 80% of individuals aged 65 years or older show radiological symptoms of KOA [1]. Pain is the most significant and widespread symptom of KOA. International recommendations for KOA treatment consist of pain alleviation and physical interventions via a combination of pharmacological and nonpharmacological therapies, such as massages [2,3]. Tuina, a massage remedy built on Chinese Medicine meridian theories and Zang-Fu organs, consolidates contemporary scientific knowledge, including anatomy, biomechanical role, and physiology. Previous randomized controlled trials (RCTs) have supported the effectiveness of Tuina for the treatment of different health conditions, such as acute bronchitis [4] and musculoskeletal disorders [5,6]. In relation to KOA, 2 systematic reviews have identified positive clinical effects of Tuina in improving the outcomes of patients with KOA [7,8]. Furthermore, our recent RCT demonstrated the safety and effectiveness of Tuina for improving pain, increasing joint flexibility, and improving KOA-related disability [9].

The pathogenesis of KOA is complex and involves heterogeneous factors in the central and peripheral nervous systems. However, peripheral candidate mechanisms are insufficient to explain widespread pain and the range of concomitant symptoms; thus, centrally driven mechanisms are deemed pivotal in the etiology of KOA. Our recent research demonstrated that the brain also participates in the pathophysiological changes in KOA and that central nervous system remodeling and function modifications may be among the central mechanisms associated with KOA [10]. Patients who reported higher levels of arthritic pain had greater opioid receptor availability in the periaqueductal gray (PAG), a key region of the descending pain inhibitory system, suggesting the alteration of this system in KOA [11]. Additionally, a functional magnetic resonance imaging (fMRI) trial demonstrated that the degree of PAG activation provides a sensitive and objective assessment of patellofemoral pain pressure in patients with KOA [12]. The descending pain inhibitory system originates in the PAG of the midbrain conduction duct and exerts its analgesic function through the relay of the rostral ventromedial medulla (RVM), inferior projection neuron of the cephalic medulla, and spinal-thalamic projection neurons in the superficial dorsal horn of the spinal cord [13]. Moreover, the subcortical structures and multiple cortices of the brain, comprising the anterior cingulate cortex, amygdala, insula, and hypothalamus, are also involved in pain modulation via the descending pain inhibitory system. The function of the descending pain inhibitory system in pain modulation and the management of the same have been substantially referenced [14,15].

We have, therefore, designed a single-center, parallel RCT to investigate the implications of Tuina and health education on KOA pain, resting-state functional connectivity (rsFC) of the cardinal regions of the descending pain inhibitory system (RVM and PAG), modifications of the voxel-based morphometry (VBM) of RVM and PAG, and behavioral alterations and their connections. We hypothesized that all treatments would relieve knee pain and share common pathways and that pain relief would be associated with the descending pain inhibitory system.

## Methods

### Trial Design

Patients will be recruited through advertisement in the Tuina clinic of the Third Affiliated Hospital of Henan University of Chinese Medicine, Zhengzhou, China. We will follow the SPIRIT (Standard Protocol Items: Recommendations for Interventional Trials) 2013 statement [16] to guide the reporting and development of our protocol. All eligible participants will be required to sign an informed consent form and will be notified that the trial will not contain any collection of human biological specimens.

### Eligibility Criteria

The inclusion criteria are as follows: (1) met the classification and reporting of KOA, per the 1986 American College of Rheumatology criteria [17]; (2) age between 40 and 70 years; (3) experienced knee pain for ≥3 months; (4) knee pain score is ≥3 on the Numerical Rating Scale (NRS) [18]; and (5) radiologically confirmed KOA (Kellgren and Lawrence [K-L] score of 2 or 3) [19].

### Exclusion Criteria

The exclusion criteria are as follows: (1) knee pain associated with other diseases, (2) the individual has undergone intra-articular injections within the last 6 months, (3) a history of knee surgery, (4) psychiatric disorders or chronic severe or acute organic diseases, (5) bleeding disorders, and (6) magnetic resonance imaging (MRI) contraindications, such as phobias or pacemakers.

### Exit Criteria and Management

Exit criteria are as follows: (1) request by the participant and (2) intratreatment side effects.

### Sample Size

The sample size of 64 patients was planned, based on our previous clinical research [9]. The formula is determined:



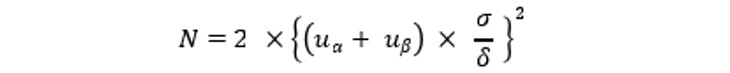



wherein *N* refers to the sample size in each group, *σ* represents the SD, *α* represents type I error, *β* represents type II error, 1–*β* represents the test power, and *δ* represents the difference between mean groups.

The pressure pain threshold (PPT) values of the treatment and control groups were 9.53 (1.26) and 9.02 (2.62), respectively. We took *α*=0.05, *β*=0.10, 1–*β*=0.90, set the 2-sided test level *α*=0.05, and found out from the *t*-distribution boundary value table: *u*_α_=1.96, *u*_β_=1.282. In this study, *δ*=0.51, *σ* with the larger SD of 1.36, and an approximate *N* of 29 after substituting the formula. With an estimated drop-off rate of 10%, a total of 64 patients with KOA were needed.

### Recruitment Strategies and Enrollment

**Figure 1:**
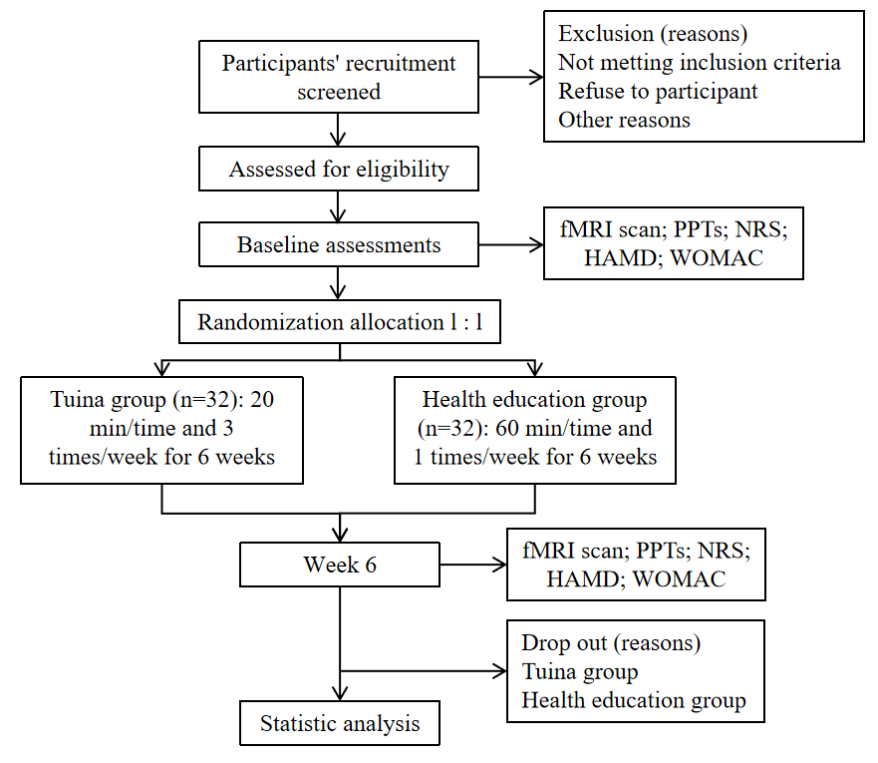
Flowchart of the trial. fMRI: functional magnetic resonance imaging; HAMD: Hamilton Depression Scale; NRS: Numerical Rating Scale; PPT: pressure pain threshold; WOMAC: Western Ontario and McMaster Universities Osteoarthritis Index.

The participants will be recruited between December 2023 and December 2024. Participants will also be recruited through a multi-modal strategy, including dissemination through WeChat and posters at community service centers. Figure 1 presents a flow diagram consisting of recruitment, screening, and randomization. Table 1 presents a schematic overview of our trial designs, conduct, review, and analysis.

**Table 1:** Chart of the trial’s time points.

Research process	Enrollment	Allocation	Beginning	End of the experiment
Eligibility screen	✓			
Demographic variables	✓			
Medical variables	✓			
Allocation		✓		
Consent			✓	
fMRI^a^ scan			✓	✓
PPTs^b^			✓	✓
NRS^c^			✓	✓
HAMD^d^			✓	✓
WOMAC^e^			✓	✓
Adverse events			✓	✓
Working practice record			✓	✓

^a^fMRI: functional magnetic resonance imaging.

^b^PPT: pressure pain threshold.

^c^NRS: Numerical Rating Scale.

^d^HAMD: Hamilton Depression Scale.

^e^WOMAC: Western Ontario and McMaster Universities Osteoarthritis Index.

### Randomization

Independent research staff will use SPSS 25.0 (IBM Corp) to generate a randomly numbered sequence for complete randomization. Participants will be randomly assigned into 1 of 2 groups (ie, health education or Tuina group) and applied in a 1:1 allocation ratio. The random sequence will be sealed by a self-governing research assistant via an opaque envelope that contains information regarding the participant’s allocation.

### Blinding

The outcome evaluators and data statisticians will all be masked to the group assignment to prevent bias.

### Interventions

A panel of 2 clinical medical professionals with an academic background in Tuina and clinical medicine agreed to develop a standard protocol for Tuina and health education. The protocol’s feasibility will be examined to determine whether it is practicable and acceptable and to identify any realistic issues caused by the procedures. Given the nature of the treatment, participants will be informed that the trial will be without harm, compensation, and posttrial care.

Clinical medical professionals with ≥3 years of experience with Tuina will conduct the health education for the participants. Before the trial, clinicians will be trained to ensure standard procedures and compliance with treatment protocol. All participants in the Tuina group will receive three 20-minute sessions a week over a 6-week treatment course. The participants in the health education group will participate in health education sessions lasting 1 hour each (administered once weekly for 6 weeks). The participants are to maintain their dietary habits and regular physical activity throughout the study period. Throughout the trial, they will be informed that they are not permitted to entertain other remedies. To guarantee compliance, all participants are required to register for therapy in a written log.

### Tuina Group

**Figure 2:**
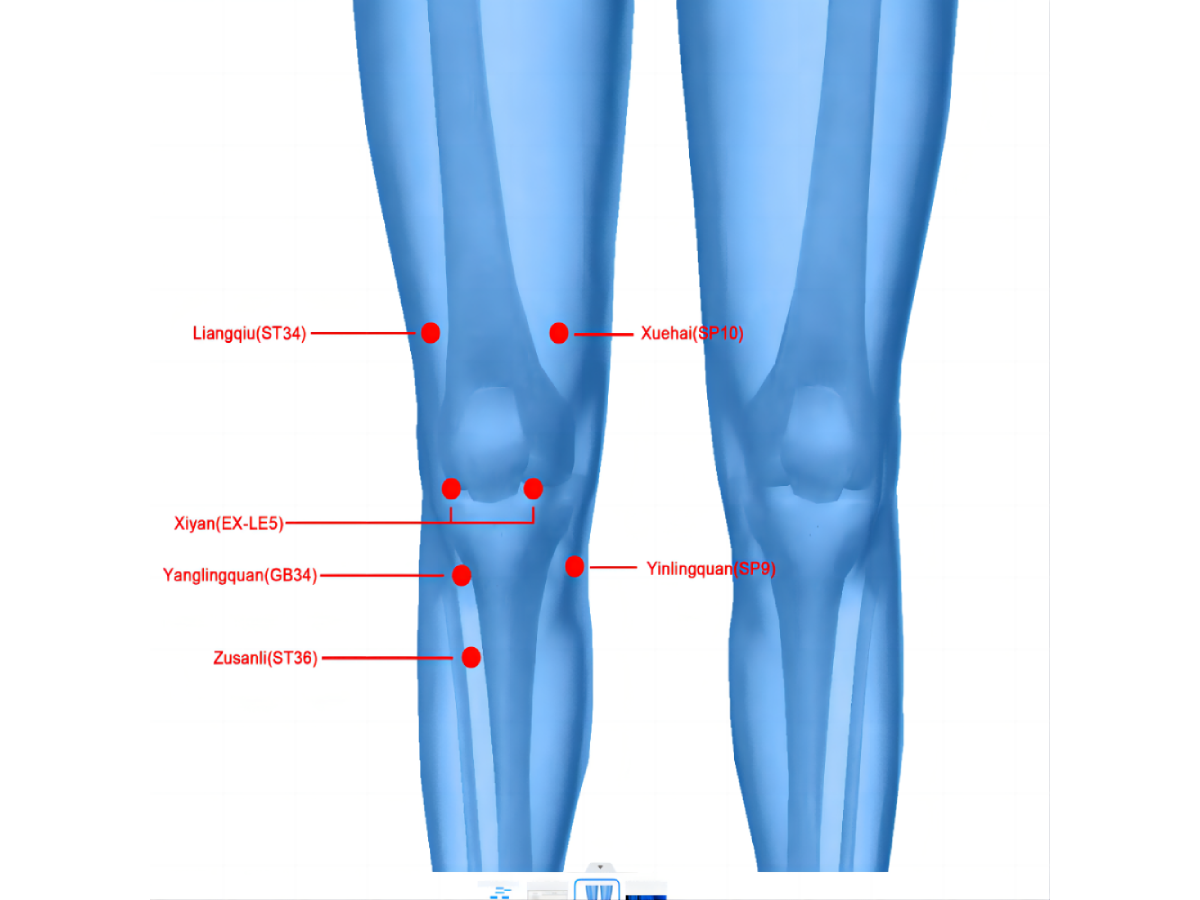
Location of the acupoints.

The standard Tuina procedure will be performed based on Tuina Therapy, a textbook of the 13th Five-year Plan [20]. The specific administration procedure will first start with the hand disinfection of the Tuina clinical medical professional. Second, the specific application of Tuina will be performed according to the following methods [9]: (1) the participant will be placed supine on the treatment bed, and the Tuina therapist will press at the points Xuehai (SP10), Yinlingquan (SP9), Yanglingquan (GB34), Liangqiu (ST34), Xiyan (EX-LE5), and Zusanli (ST36; based on the standard of the World Health Organization [21]; Figure 2); (2) the participant will be asked to sit in a chair, the knee will be flexed to 90 degrees, and the Tuina therapist will revolve the foot of the participant to a neutral position and lightly press the patella’s lower edge; the therapist’s other fingers will then be wrapped around the knee’s rear without force. The participant will be instructed to stand, and the therapist will push the patella. The participant will then be required to sit down. This process will be repeated 3 times.

### Health Education

We will introduce the basic concepts of KOA and the methods used to manage the associated risks to help participants protect their knees.

### MRI Data Acquisition

MRI will be performed at the MRI Center of The Third Affiliated Hospital of Henan University of Chinese Medicine. Resting-state fMRI will be performed at the beginning and end of the experiment. MRI data will be acquired using a 3.0-T magnetic resonance scanner (General Electric) with a 32-channel phase-array head coil. During the full scanning period, the participants will be asked to remain awake and motionless with their eyes closed.

Blood oxygen level independent resting-state functional images will be acquired using the following parameters: TR=2000 ms, TE=30 ms, flip angle=90°, 33 axial slices, and field of view (FOV) = 220 mm × 220 mm. T1-weighted images will be collected using the following parameters: 160 axial slices, TE=2.93 ms, flip angle=9°, TR=1900 ms, TE=30 ms, and FOV = 256 mm × 256 mm. T2-weighted images will be collected with the following parameters: 25 axial slices, TE=2.93 ms, flip angle=150°, TR=6300 ms, TE=86.0 ms, and FOV = 240 mm × 240 mm.

### Clinical Outcome Assessments

Validated pain detectors and questionnaires will be used to assess knee pain, psychological characteristics, and function at the beginning and end of the experiment. The clinical outcome assessments will be (1) the PPTs, (2) the NRS, (3) the Hamilton Depression Scale (HAMD), and (4) the Western Ontario and McMaster Universities Osteoarthritis Index (WOMAC). Additional outcome indices included the adverse events of treatment.

### Pressure Pain Thresholds

**Figure 3:**
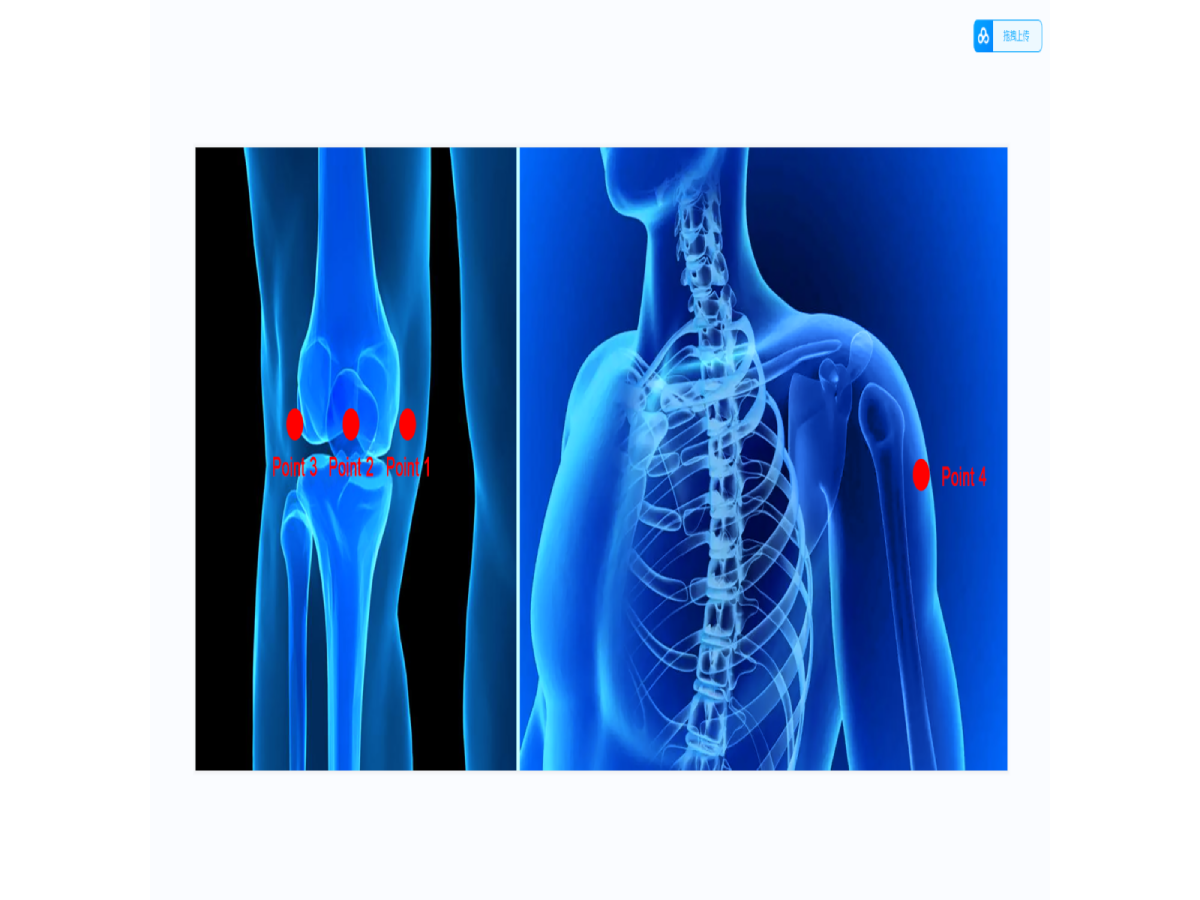
Points used to measure the PPTs. PPT: pressure pain threshold.

The PPT is a form of quantitative sensory testing that enhances the understanding of pain sensitivity and musculoskeletal pain [22-24]. Moreover, it has strong interrater reliability across multiple raters [25]. The PPT effectively depicts pain intensity and is widely applied in patients with KOA. PPTs will be measured using a portable machine (FDX50, Digital Force Gauge, Wagner Instruments) [26,27]. The patient will be placed in a supine position. The variable collector will select four points for measurement: (1) 3 cm medial to the midpoint of the medial patellar border, (2) the center of the patella, (3) 3 cm lateral to the midpoint of the lateral patellar border, and (4) the belly of the deltoid muscle of the affected upper limb (Figure 3) [28]. The pain test needle will be moved slowly at 0.1 kg/s until the participant feels pain. The measurement will be repeated 3 times at each site with an interval of 25 s, and the average will be calculated [29].

### Numerical Rating Scale

The NRS is an elementary well-managed scale to assess pain intensity. Patients express the intensity of rest and movement pain using the numbers 0-10.

### Hamilton Depression Scale

The HAMD comprises 24 items, most of which are evaluated on a 5-point scale (0-4 points): 0=“none,” 1=“mild,” 2=“moderate,” 3=“serious,” and 4=“very serious.” A few items are assessed on a 3-point scale (0-2 points): 0=“none,” 1=“mild to moderate,” and 2=“serious.”

### Western Ontario and McMaster Universities Osteoarthritis Index

The WOMAC (0-10 cm) comprises 3 dimensions (ie, pain, stiffness, and joint function). The score ranges from 0 to 96, with higher scores indicating worse symptoms.

### Adverse Events of Treatment

All adverse events that may occur throughout the trial will be documented, including the time of occurrence, symptoms of discomfort, particular indications, severity, remedy supplied, time course of the action of amelioration, time of determination, and date of remedy termination.

### Demographic or Medical Variables

The following demographic and medical variables will be collected for analysis: sex, age, marital status, occupation, ethnicity, education level, blood pressure, temperature, respiration, pulse, height, weight, body mass index, combination of disease and medication, K-L score, course of KOA, and history of major surgeries.

### Data Management

Clinical data will be carefully saved using printed and electronic case report forms (CRFs). Only outcome assessors will access the CRFs for data entry to guarantee data quality. CRFs will be verified for double entry and accuracy. During the trial, the Third Affiliated Hospital of the Henan University of Chinese Medicine will conduct regular visits (once a week) to review the conduct of the trial.

### Data Monitoring

The ethics committee will monitor for trial breaches and guarantee no conflicts of interest exist. Statisticians can obtain the last trial data set, which will exclusively accommodate coded data. The safety, advancement, and research integrity will be supervised throughout the research team conferences.

### fMRI Preprocessing

#### rsFC Preprocessing

rsFC will be conducted by applying a seed-based approach using the CONN toolbox (Version 17.f) [30]. CONN is Matlab/statistical parametric mapping (SPM)–based software for the analysis of functional connectivity data. This analysis will use predefined regions of interest (ROIs) based on masks of the a priori specified brainstem nuclei (PAG and RVM) [31], as previously described [32].

Preprocessing of fMRI data will be performed using a pipeline in the CONN toolbox, including translation by centering to (0, 0, 0) coordinates, slice-timing correction, realignment, coregistration to participants’ respective structural images, normalization, artifact detection, smoothing with an 8 mm Gaussian kernel, and segmentation into gray matter volume (GMV), white matter (WM), and cerebrospinal fluid volume (CSF). Linear detrending will be performed with a frequency window of 0.008-0.09 Hz. Linear detrending will be executed, and a frequency window of 0.008-0.09 Hz will be applied. Artifact detection will be run using the ART toolbox to eliminate correlations caused by head motion and artifacts. During the denoising processes, WM, CSF, and outliers will be detected using the ART toolbox and entered into the linear regression analysis as confounding effects.

Subsequently, a correlation map will be produced for each participant by extracting the blood oxygen level independent time course from the left/right PAG and left/right RVM seeds separately and computing the Pearson correlation coefficients between the time course in the PAG or RVM and every voxel of the whole brain. Correlation coefficients will be converted by applying Fisher transformation into *z* scores to raise their normality.

#### VBM Preprocessing

We will use the Diffeomorphic Anatomical Registration With Exponentiated Lie Algebra (DARTEL) SPM 12’s segmentation algorithm for whole brain VBM analysis. All T1-weighted images will be analyzed using SPM 8 (Wellcome Institute) software, as implemented in Matlab (Matlab 2013a, Math Works). First, the new segmentation algorithm from SPM 8 will be applied to every T1-weighted image will be segmented into GMV, WM, and CSF. Second, all segmented tissue maps will be used to create a vervet population template using the DARTEL template-creation tool. We will use a set of standard Montreal Neurological Institute tissue maps and a multivariate tissue-affinity-registration and segmentation algorithm carried by SPM’s VBM DARTEL for that process. Finally, each patient’s GMV map will be warped using its corresponding smooth (Gaussian kernel of 8 mm full-width at half maximum), reversible martensitic parameters to transform it to the customized template space and then to the Montreal Neurological Institute template space.

### Statistical Analysis

Prior to the analysis, a statistical program will be undertaken by a statistician. The program will embrace the processing approaches and the demanded data. Variables will be analyzed with IBM SPSS Statistics for Windows (version 25; IBM Corp).

### rsFC Analysis

For the ROI analysis, the ROI-to-ROI connectivity connections will be placed to a threshold by the intensity at the false discovery rate-corrected *P*<.05 (2-sided).

For the analysis of rsFC, we will use ROIs derived from the rsFC results. For the ROIs, we will use a threshold of voxel-wise *P*<.005 uncorrected and *P*<.05 corrected using Monte Carlo simulations with the 3dFWHMx and 3dClustSim applied [33].

### VBM Analysis

After completing these image analyses, we will obtain the GMV values of the key regions derived from rsFC for subsequent statistical analysis. The statistical significance between-group differences will be set at *P*<.05 with family-wise error correction.

### Data Analysis of Clinical Outcomes

#### Measurement Data

The Shapiro-Wilk test will foremost be conducted to confirm the normality of the uninterrupted data distribution. Independent sample *t* tests will be conducted for normally distributed data to evaluate the baseline characteristics. The Mann-Whitney U test will evaluate between-group differences for nonnormally distributed data.

#### Ranked Data and Count Data

The body mass index categorization, K-L score, NRS score, and WOMAC score are the rank data. Therefore, the Mann-Whitney U test will be applied for the between-group comparisons, and the Wilcoxon symbolic test will be applied for the intragroup comparisons. Count data will involve the ratio of the sex variable and knee constituent ratio, compared between the 2 groups via *χ*^2^ test of 4-fold table or Fisher test. Statistical significance will be set at *P*<.05 (2-sided).

### Ethical Considerations

This trial was registered in the Chinese Clinical Trials Registry in 2023 (ChiCTR2300070289). The ethics committee of the Third Affiliated Hospital of Henan University of Chinese Medicine has approved this study protocol (ethics approval number 2022HL-031).

This RCT will adhere to the ethical principles governing biomedical research on human participants and respect fundamental human rights in accordance with international recommendations included in the Declaration of Helsinki and its subsequent revisions.

Participants will be given both written and oral information about why the trial will be conducted, its objectives, the potential risks associated, and the guidelines set out during the monitoring period. If a patient fulfills the inclusion criteria and agrees to enroll in the trial, they will provide written informed consent. Patients are free to withdraw consent and discontinue the trial at any time and for any reason.

## Results

This trial was approved by the ethics committee of the Third Affiliated Hospital of Henan University of Chinese Medicine. Enrollment began in December 2023, and the results of this trial are expected to be submitted for publication in May 2025.

## Discussion

### Overview

To the best of our knowledge, our trial will be the first to assess the modulatory effect of Tuina on the descending pain inhibitory system in patients with KOA. The results will improve the apprehension regarding Tuina’s mechanisms in patients with KOA.

KOA can cause joint dysfunction, reduce physical fitness and capability to tackle activities of daily living, and cause depression and anxiety [3,34,35]. Previous researchers have indicated that peripheral factors such as synovial inflammation and articular cartilage devastation may have a cardinal role [36-38]. However, many studies found that the severity of radiographic KOA and the inflammatory response of the synovial membrane do not positively correlate with KOA [39-41]. Consequently, peripheral mechanisms cannot completely explain KOA. Patients with KOA have simultaneously raised pain sensitivity in affected and nonaffected areas. Therefore, some investigators suggest that an “abnormal central processing” of afferent pain messages may be a cardinal factor in KOA [26].

Based on the theory of traditional Chinese Tuina, all the acupoints can treat the disease of the adjacent area and local area. In our study, 6 local acupoints are selected in the Tuina group, including Xuehai (SP10), Yinlingquan (SP9), Yanglingquan (GB34), Liangqiu (ST34), Xiyan (EX-LE5), and Zusanli (ST36). Yanglingquan (GB34) and Yinlingquan (SP9) are also known as the converging point of the tendon footprint and are usually recommended to relieve knee pain. Xuehai (SP10) and Liangqiu (ST34) are the most commonly used acupoints for the treatment of dysfunction caused by KOA. Xiyan (EX-LE5) is supplemented to adjust the tension of soft tissue. Zusanli (ST36) is commonly used for the treatment of inflammation and pain. Our previous results showed that the above 6 acupoints used together can effectively improve the pain of patients with KOA [7].

Moreover, Tuina is safe and effective for pain, physical function, stiffness, and other clinical symptoms associated with KOA [42]. It likewise has been demonstrated to activate and modulate functional connectivity in the hippocampus to facilitate cognitive function in patients with poststroke depression [43]. The brain has a powerful control over nociceptive input in the spinal cord at the brain stem level. This top-down control occurs through the “descending modulation” of the pain transmission pathway mechanism [44-46]. The descending pain inhibitory system has a cardinal role in normal pain consciousness, and its malfunction may be one of the pathophysiological mechanisms in KOA. This factor possibly clarifies how other centrally mediated processes, such as sleep, mood, the placebo effect, and cognition, influence the pain experience [47]. Crucially, the PAG and RVM, as important components of the descending pain inhibitory system, directly modulate the activity of the spinal neurons involved in pain transmission [48]. Furthermore, animal experiments have demonstrated that the PAG is involved in opioid-mediated analgesia since the microinjection of morphine into this nucleus produces a reduction in sensory pain behaviors [49]. Similarly, the analgesic effects of stimulating the PAG directly have also been evidenced in humans [50]. Moreover, direct PAG stimulation is used to diminish chronic pain intensity in individuals [51]. The RVM is the pontomedullary area where opioid microinjection generates analgesia [48]. Making allowances for this, the PAG-RVM axis, as a significant component of the descending pain inhibitory system, can possess an inhibitory implication on harmful transmission. However, the mechanism of the descending pain inhibitory system in the analgesic effect of Tuina in the treatment of KOA is unclear, especially the relationships among PAG and RVM and clinical behavioral indicators, which limits its promotion and application. Therefore, fully exploring the central analgesic mechanism is necessary to alleviate the pain of KOA. As a main type of fMRI, resting-state fMRI has the advantage of providing more comprehensive information on the functional architecture of the brain. Resting-state fMRI is widely used to study the analgesic mechanism of Tuina in the treatment of KOA [52].

In summary, this clinical trial will aim to investigate the influence of Tuina on the descending pain inhibitory system in order to explain the potential central mechanism of Tuina treatment. We will use a combination of resting-state MRI and clinical behavior indicators such as rsFC and VBM to perform research and analysis of Tuina analgesia.

### Limitations and Strengths

Although this study’s potential strengths, it anticipates limitations. First, there is potential for methodological bias, as blinding is particularly difficult when delivering a physical intervention or health education. Second, the long-term implications of Tuina will not be investigated; thus, further research is warranted. Despite these several different limitations, this study’s strengths lie in its focused investigation of the potential central mechanism of Tuina treatment, which contributes valuable insights into the modulatory effect of Tuina on the descending pain inhibitory system in patients with KOA.

### Conclusions

This trial is designed to investigate the central mechanism of Tuina in the treatment of KOA, with the involvement of health education as a control intervention. We expect that Tuina would relieve pain symptoms in patients with KOA and that pain relief would be associated with the descending pain inhibitory system.

## Abbreviations

CRF: case report form
CSF: cerebrospinal fluid volume
DARTEL: Diffeomorphic Anatomical Registration With Exponentiated Lie Algebra
fMRI: functional magnetic resonance imaging
FOV: field of view
GMV: gray matter volume
HAMD: Hamilton Depression Scale
K-L: Kellgren and Lawrence
KOA: knee osteoarthritis
MRI: magnetic resonance imaging
NRS: Numerical Rating Scale
PAG: periaqueductal gray
PPT: pressure pain threshold
RCT: randomized controlled trial
ROI: regions of interest
rsFC: resting-state functional connectivity
RVM: rostral ventromedial medulla
SPIRIT: Standard Protocol Items: Recommendations for Interventional Trials
SPM: statistical parametric mapping
VBM: voxel-based morphometry
WM: white matter
WOMAC: Western Ontario and McMaster Universities Osteoarthritis Index

## Data Availability

The data sets used and analyzed during this study are available from the corresponding author upon reasonable request.
